# A novel tertiary lymphoid structure-associated signature accurately predicts patient prognosis and facilitates the selection of personalized treatment strategies for HNSCC

**DOI:** 10.3389/fimmu.2025.1551844

**Published:** 2025-03-13

**Authors:** Jinhao Zhang, Lu Zeng, Guobin Song, Gaoge Peng, Zhezheng Chen, Yamin Yuan, Taowu Chen, Tao Zhong, Shixi Chen, Zhengzhou Luo, Jingang Xiao, Lin Liu

**Affiliations:** ^1^ School of Stomatology, Southwest Medical University, Luzhou, Sichuan, China; ^2^ Department of Oral and Maxillofacial Surgery, The Affiliated Stomatological Hospital, Southwest Medical University, Luzhou, China; ^3^ Luzhou Key Laboratory of Oral & Maxillofacial Reconstruction and Regeneration, Southwest Medical University, Luzhou, China; ^4^ Key Laboratory of Breast Cancer in Shanghai, Fudan University Shanghai Cancer Center, Shanghai, China; ^5^ Department of Oncology, Shanghai Medical College, Fudan University, Shanghai, China

**Keywords:** tertiary lymphatic structure, HNSCC, GSVA, tumour microenvironment, immunotherapy, nomogram, prognostic signature

## Abstract

**Background:**

Head and neck squamous cell carcinoma (HNSCC) is the most common type of head and neck cancer and is characterized by its aggressive nature and variable prognosis and response to immunotherapy. Tertiary lymphoid structures (TLSs) play crucial roles in creating a favourable immune microenvironment to control tumour progression. However, the specific impact of these structures on HNSCC has not been thoroughly studied.

**Methods:**

In this study, a comprehensive review of tertiary lymphoid structures was conducted by analysing 9 TLS-associated genes in a cohort of 871 HNSCC patients. Distinct TLS-related subgroups were identified through unsupervised clustering analysis, and the associated genes were explored. Prognostic genes were identified via univariate Cox and Boruta algorithms, and a novel TLS-related scoring system was developed via the GSVA algorithm.

**Results:**

Our study revealed that patients with higher TLS-related scores had improved overall survival and were more likely to benefit from immunotherapy. Furthermore, we observed a significant negative correlation between sensitivity to traditional chemotherapeutic agents and the TLS-related signature score.

**Conclusions:**

Our findings suggest that the TLS-related features of HNSCC patients hold promise as predictive indicators for immunotherapy efficacy and may offer novel insights for tailoring personalized treatment strategies in clinical practice.

## 1 Introduction

Head and neck cancer, the seventh most common cancer globally, primarily affects areas such as the oral cavity, mouth, nasopharynx, larynx, and hypopharynx, with squamous cell carcinoma being the predominant type. Approximately 600,000 new cases of head and neck cancer are reported annually worldwide, with a 5-year survival rate of only 40–50%. In 2020, there were 931,931 new cases and 467,125 deaths worldwide. The histological grade and TNM stage play crucial roles in determining the prognosis of HNSCC patients and can guide treatment decisions, such as prognostic grading, immunotherapy, and radiotherapy (1, 2). However, variations in clinicopathologic features among HNSCC patients suggest limitations in traditional staging methods, highlighting the need for new prognostic biomarkers to improve patient outcomes. Given the tendency of HNSCC to metastasize to local lymphatic vessels, addressing tumour-draining cervical lymph nodes is essential for effective treatment.

Tertiary lymphoid structures (TLSs) are organized aggregates of immune cells that develop in various nonlymphoid tissues and organs, resembling secondary lymphoid organs (SLOs) (3, 4). As TLSs mature, they promote the formation of hyperendothelial small veins (HEVs) and the lymphatic vascular system, enhancing immune cell recruitment (5). The microenvironment can either promote tumour growth, invasion, and metastasis or inhibit tumour progression by triggering a successful antitumour immune response. For example, in non-small cell lung cancer patients, the presence of TLSs near the lung tumour correlates with a significantly better overall prognosis and long-term survival than the absence of TLSs. The presence of TLS in melanoma has been linked to improved responses to immunotherapy, suggesting their potential as predictive biomarkers. The maturation of TLS, characterized by well-developed germinal centres, enhances their tumour-suppressive capabilities, further emphasizing their importance in sarcoma prognosis (6, 7). Studies indicate that the densities of T cells, dendritic cells, B-cell follicles, and germinal centres within tumour-associated TLSs are associated with improved clinical outcomes, suggesting a functional connection between the development of tumour-associated TLSs and protective responses against tumours.

In this study, a consensus unsupervised clustering approach was used to classify HNSCC patients into different clusters on the basis of nine tertiary lymphoid structure-associated genes known to affect prognosis. By integrating data from three HNSCC cohorts, univariate Cox analysis and the Boruta algorithm were utilized to narrow down the pool of differential genes among TLS clusters. Following the identification of candidate genes, a novel scoring system was developed on the basis of the GSVA algorithm. This study aimed to assess the utility of newly constructed TLS-related signatures in stratifying patient risk, assisting clinicians in devising effective immunotherapies and identifying individuals who may be more responsive to treatment.

## 2 Method

### 2.1 Access to raw data

RNA-seq data and corresponding clinical information for HNSCC patients were obtained from the UCSC Xena (https://xena.ucsc.edu/) and GEO (http://www.ncbi.nlm.nih.gov/geo/) databases, respectively. The TCGA cohort consisted of 504 tumour patients and 44 normal controls. The genetic profiles and clinical data of 367 HNSCC patients in the GSE41613 and GSE65858 datasets were downloaded from the GEO database. Quality control checks were performed on each dataset to ensure consistency and reliability. Gene expression levels were normalized using appropriate normalization methods. The datasets (GSE41613 and GSE65858) were integrated by aligning gene identifiers across datasets. The mRNA expression data from these cohorts were merged and batch corrected via the “sva” package. The sva package from Bioconductor was used for batch correction. Surrogate variables were estimated to account for technical variability introduced during the sequencing process. The ComBat function from the sva package was employed to adjust for batch effects. Principal Component Analysis (PCA) was performed to visualize the effect of batch correction. The first few principal components should show reduced variation between batches after batch correction. In addition, 9 TLs-associated genes were extracted from a recent Nature article on tertiary lymphoid structures (8) for further analysis.

### 2.2 Consensus unsupervised clustering method

To identify TLS-related clusters, we used the software program “ConsensusClusterPlus” (9) to analyse the expression of the 9 TLs-associated genes. The optimal k value was determined on the basis of the proportion of fuzzy clusters (PAC), which was indicated by the lowest PAC value. We subsequently conducted differential expression analysis of genes within various TLS clusters via the “limma” package, with criteria of |log2 FC|>1 and adjusted P values <0.05.

### 2.3 Functional enrichment analysis

We performed “GO” enrichment and “KEGG” pathway analysis to investigate the biological functions of specific genes that were differentially expressed in various TLS-associated clusters. The “clusterProfiler” package was used for this analysis, with the “BH” method applied for P value adjustment (10). The GO enrichment analysis covered cellular components, biological processes and molecular functions.

### 2.4 Development and validation of a new scoring system for TLS features

A secondary clustering analysis was performed on differentially expressed genes (DEGs) within various TLS clusters. Following one-way Cox analysis of these DEGs associated with TLS gene clusters, two gene signatures were defined: gene signature A for genes positively associated with TLS gene clusters and gene signature B for genes negatively associated with TLS gene clusters. The Boruta algorithm, implemented through the ‘Boruta’ software package with 500 maxRuns, was used to further filter out significant genes among the candidate genes. The genes identified as “confirmed” genes were retained. The characteristic scores of these significant gene sets in signature A and signature B were subsequently calculated via the “GSVA” method. A new TLS-related scoring system was established using the formula TLS score= GSVAA -GSVAB. The optimal cut-off value was determined via the “surv_cutpoint” function in the “survminer” package. HNSCC patients were then classified into high and low subgroups on the basis of the optimal cut-off value.

### 2.5 Quantification of the tumour immune microenvironment by TLS features

Currently, commonly used immune cell infiltration algorithms include XCELL (11), TIMER (12), QUANTISEQ, MCPCOUNT, EPIC (13), CIBERSORT (14) and CIBERSORT-ABS. Spearman correlation analysis was used to examine the associations between immune cells and risk scores in HNSCC patients. Using the single-sample GSEA (ssGSEA) method, patients with low TLS scores were separated from those with high scores on the basis of immune cell characteristics. The tumour microenvironment was evaluated via the R program “ESTIMATE” to combine estimated scores. The immune score and interstitial score were used to assess the immune and interstitial components of the tumours in each HNSCC sample.

### 2.6 Development and validation of a new TLS signature

This study assessed the predictive value of the TLS score in determining immunotherapy response in HNSCC patients via the Tumour Immune Dysfunction and Rejection (TIDE) algorithm (15). The validation cohort GSE79671 included 16 HNSCC patients treated with bevacizumab combination therapy, with 6 patients showing a positive response to immunotherapy. The GSVA approach was used to calculate the immune cycle associated with cancer and immunotherapy, with enrichment scores calculated for favourable genetic characteristics, and a p value of 0.05 was considered statistically significant between the two groups. The relationships between risk scores and the aforementioned genetic traits were analysed via the R package “ggcor”.

### 2.7 Drug sensitivity analysis

To predict and validate the potential of the TLS profile in targeted chemotherapy, “pRRophetic” was used to calculate the IC50 values of various chemotherapeutic drugs used for the treatment of HNSCC. By comparing the IC50 values between the high and low subgroups, we evaluated the sensitivity of patients to each drug.

### 2.8 scRNA-seq processing

The GSE103322 dataset was obtained from the GEO database (https://www.ncbi.nlm.nih.gov/geo/). Preprocessing of the single-cell RNA sequencing (scRNA-seq) data was performed via the ‘Seurat’ package. The PercentageFeatureSet function was employed to determine the proportion of mitochondrial genes. Correlation analysis was conducted to explore the relationships among sequencing depth, mitochondrial gene sequences, and total intracellular sequences. Each gene was required to be expressed in at least 5 cells. Cells were retained on the basis of specific criteria, including gene expression levels ranging from more than 300 to less than 5000, a mitochondrial content less than 10%, and a unique molecular identifier (UMI) count greater than 1000. Following data filtering, the scRNA-seq data were normalized via the log normalization method. The “CellCycleScoring” function was used to compute S and G2M scores via gene expression data associated with the S phase and G2M phase, respectively. This analysis confirmed the phase of each cell as S phase, G2M phase, or G1 phase. Additionally, the “copykat” package in R was used to predict the copy number variation of each cell, aiding in distinguishing between diploids (normal cells) and aneuploids (tumour cells).

### 2.9 Spatial transcriptomics

The sequencing information of HNSCC patients was extracted from the GEO dataset GSE144240 (https://www.ncbi.nlm.nih.gov/geo/query/acc.cgi?acc=GSM4284256) and analysed via the Seurat package for quality control of spatial transcriptomics data. Data normalization was carried out via the SCTransform function, followed by unsupervised clustering with Seurat. Integration of snRNA-seq and spatial transcriptomics data involved measuring the degree of overlap in expression levels between cell type-specific genes identified by snRNA-seq data and area-specific genes characterized by spatial transcriptomics data via the MIA approach. A lower p value indicates stronger overlap. Additionally, copykat analysis was performed to predict the copy number variation of each cell.

### 2.10 Pseudotime analysis

Monocle software was used to analyse the pseudotime trajectories of single-cell subpopulations to explore changes in the process of cell differentiation (16). The raw UMI counts and their clustering information were input into the “newCellDataSet” function, after which they were transformed into a reduced dimension space via the tree discriminant reduction (DDRTree) technique, which is a widely used manifold learning method. The single-cell subpopulations were subsequently ordered on the basis of pseudotime, and pseudotime heatmaps were generated via the patchwork package to identify genes that change synchronously with pseudotime, along with cell trajectory plots.

### 2.11 Cell communication analysis

Cellcall is a tool for analysing intercellular and intracellular signalling on the basis of the KEGG ligand−receptor-transcription factor (L-R-TF) axis dataset (17). It combines the expression of ligands/receptors with the downstream transcription factor (TF) activity of certain ligand−receptor (L−R) pairs to study intercellular communication networks and internal regulatory signals. Additionally, this technique incorporates an embedded pathway activity analysis method to explore key pathways involved in cell communication. In this study, Cellcall quantified pathway activity on the basis of Jaccard similarity coefficients and assessed the significance of pathway activity through hypergeometric testing.

### 2.12 Cell culture and transfection

HOK, HSC3, SCC9, SCC15, SCC25, and CAL33 cells were purchased from ATCC and cultured in DMEM/F12 supplemented with 10% foetal bovine serum (FBS) and 1% penicillin−streptomycin. The cells were grown in a 5% CO_2_ incubator at 37°C. Lentiviral vectors containing SLC5A8 cDNA or shRNA were obtained from GeneChem (Shanghai, China) (18). To construct a lentivirus-mediated silencing vector, three shRNA sequences targeting human SLC5A8 were cloned and inserted into the hU6-MCS-CBh-gcGFP-IRES-puromycin vector. The sequence with the most effective knockdown (shRNA2 and shRNA3) was selected for further study. To knock down SLC5A8 expression, SCC9 cells were infected with lentivirus containing 4 mg/mL polybrene (19). After 48 hours, the culture medium was supplemented with 3 μg/mL puromycin to select for infected cells.

### 2.13 Quantitative real-time PCR

Total RNA was extracted via the Steadypure Quick RNA Extraction Kit (Accurate Biology, China) following the manufacturer’s instructions (20). cDNA was subsequently synthesized with HiScript III All-in-one RT SuperMix Perfect (Vazyme, China). RT−qPCR was performed via the ChamQ Universal SYBR qPCR Master Mix (Vazyme, China) on a Bio-Rad CXF96 real-time system (Bio-Rad, USA). The expression level was quantified via the 2^-ΔΔCT^ method, with GAPDH used as the reference gene for quantitative analysis. The primers used in this study were as follows: GAPDH-F: ACCCACTCCTCCACCTTTGAC; GAPDH-R: TCCACCACCCTGTTGCTGTAG; SLC5A8-F:CCTTCACATGGACCAGCATCTACG; and SLC5A8-R: GCCCAGAGTCCCACAAGATTGATG.

### 2.14 Cell proliferation and colony formation assays

Cell proliferation was assessed via the Cell Counting Kit-8 (CCK-8) (Apex Bio, USA) following the manufacturer’s protocol (21). Initially, the cells were seeded at a density of 2000 cells per well in 96-well plates and incubated at 37°C for 0, 1, 2, 3 or 4 days. Subsequently, 10 μL of CCK-8 solution was added to the cell culture medium. After a one-hour incubation, the absorbance at 450 nm was measured via a microplate reader (BioTek, USA).

For the colony formation experiments, cells were seeded into six-well plates at a density of 2000 cells per well in triplicate and cultured for 14 days. Next, the colonies were fixed with 4% formaldehyde for 15 minutes and stained with 0.1% crystal violet for 10 minutes. Then, the colonies were photographed, and those larger than 1 mm (>50 cells per colony) were counted.

### 2.15 Cell migration and invasion assays

Cell migration and invasion assays were performed using Chambers (Corning, USA) and BioCoat Matrigel Invasion Chambers (Corning, USA) (22), respectively. Briefly, cells (1×10^4^ cells/well) suspended in FBS-free DMEM/F12 culture medium were added to the upper chamber, while 1 mL of DMEM/F12 containing 10% FBS was added to the lower compartment. After a 24-h incubation, the cells remaining on the upper surface of the filter membrane were removed, and those that had crossed the filter membrane and adhered to the lower membrane surface were stained with 0.1% crystal violet. Images of the stained lower surface were captured, and the cells were quantified under a microscope in five random fields.

### 2.16 Wound-healing assay

Transfected cells were seeded into 6-well plates and cultured until they reached 95% confluence. Each well was gently scraped with a sterile 200 mL plastic pipette tip, and unattached cells and detritus were removed with PBS. Scratch wounds were photographed at 0, 24, 48 and 72 hours, and the ImageJ program was used to measure the width of the scratches.

### 2.17 Statistical analysis

All the analyses were conducted with R version 4.2.0. Kaplan−Meier plots and log-rank tests were performed to assess prognostic value and compare overall survival (OS) across subgroups within each dataset. Significance tests comparing groups utilized the Kruskal−Wallis and Wilcoxon tests. Univariate and multivariate analyses were conducted to compare the newly created TLS characteristics with other clinical characteristics to determine whether the TLS score could independently predict outcomes. Spearman correlation analysis was used to reveal the relationships between TLS-related scores and immune, stromal and estimated scores. A p value of less than 0.05 was considered statistically significant.

## 3 Results

### 3.1 Genetic and transcriptional alterations of TLSs in HNSCC

**Figure 1:**
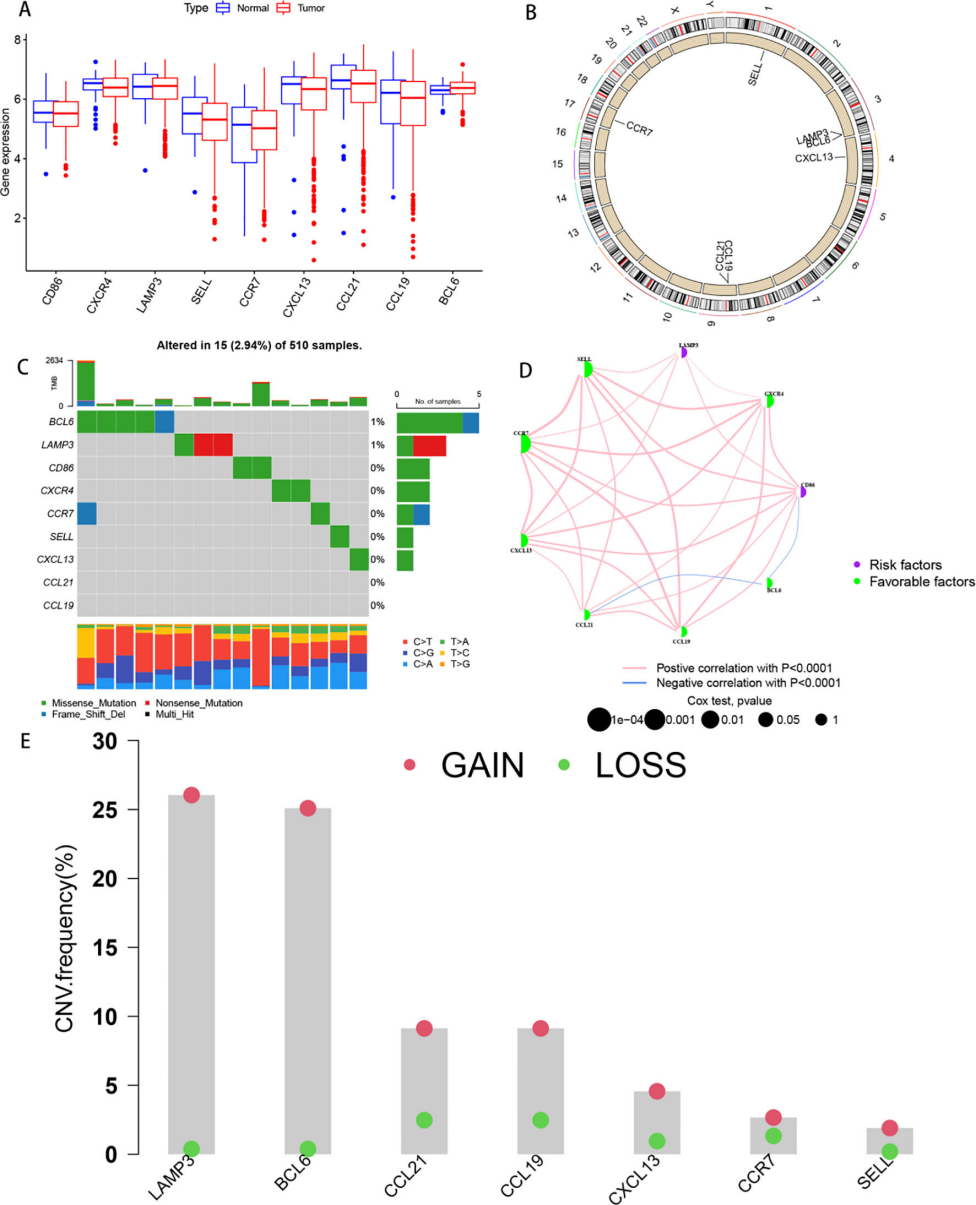
Genetic and transcriptional alterations of TLSs in HNSCC. **(A)** Box plots comparing the gene expression levels of 9 tertiary lymphoid structure-related genes in HNSCC and normal tissues from transcriptome sequencing of TCGA datasets. **(B)** Circle diagram of the locations of 9 tertiary lymphoid structure-related genes on human chromosomes. **(C)** Waterfall plot summarizing the frequency and type of mutations in nine tertiary lymphoid structure-related genes identified via methylation sequencing in 510 HNSCC patients. **(D)** Correlations between the gene expression levels of nine tertiary lymphoid structure-related genes and the correlations between genes and the prognosis of patients with HNSCC. The colour of the dots represents patient prognosis. **(E)** The bar graph shows the ordering of the frequency of copy number variations (CNVs) in the tertiary lymphoid structure-related genes of the tumour samples in the TCGA dataset; gain represents amplification, and loss represents deletion.

In total, 9 TLs-associated genes were collected, and gene expression levels were compared between HNSCC and normal tissues by integrating the expression profiles of the TCGA and GEO datasets. The results revealed differences in TLS levels between tumour and normal samples, as depicted in **Figure 1A**, with the chromosomal locations of TLs-associated genes illustrated in **Figure 1B**. A waterfall plot summarizing the frequency of somatic mutations in 510 HNSCC patients revealed a relatively low mutation frequency in 7 TLs-associated genes, with only 15 (2.94%) displaying mutations (**Figure 1C**). gene expression profiling of HNSCC samples confirmed the presence of TLSs, identified through canonical TLS signatures including lymphoid aggregates with upregulated markers such as CXCL13, CCL19, and CCL21 (23). An efferocytosis network revealed a broad topography of IRG contacts, regulator connections and their predicted correlation in HNSCC patients (**Figure 1D**). Subsequent analysis of somatic copy number changes in seven TLs-associated genes revealed a general increase in copy number variation (CNV) in LAMP3, whereas CCL19 and CCL21 showed a decrease in CNV (**Figure 1E**). The findings revealed significant differences in the gene composition and expression levels of TLs-associated genes between HNSCC and control samples, suggesting a potential role of TLSs in the development of HNSCC.

### 3.2 Correlations of TLSs with the immune microenvironment of HNSCC tumours

**Figure 2:**
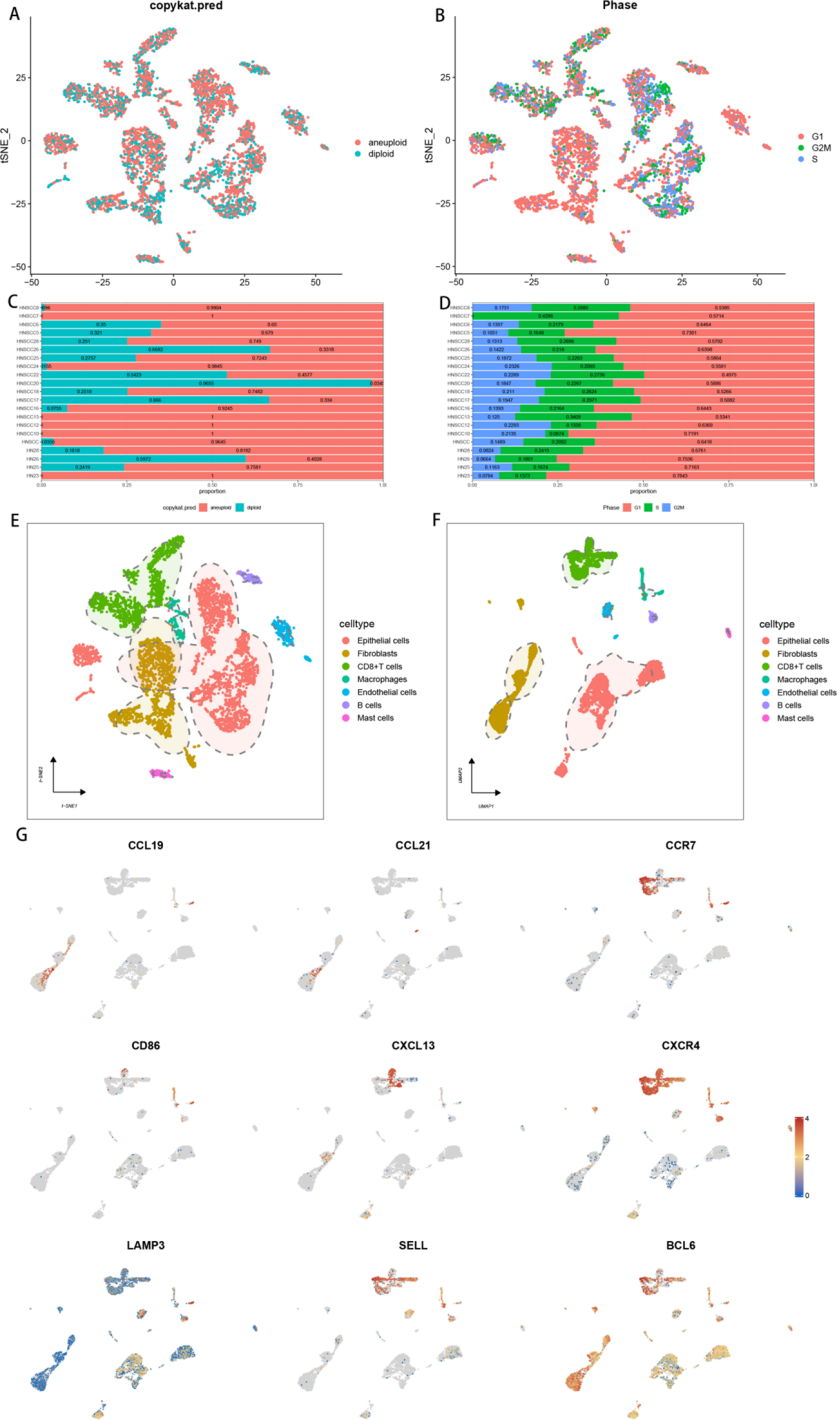
Correlation of TLSs with the immune microenvironment of HNSCC. **(A)** t-SNE plot showing the distribution of aneuploid and diploid cells. **(B)** t-SNE plot showing the distribution of cells in different phases: G1, G2M, and S. **(C)** Bar graph illustrating the proportion of aneuploid and diploid cells in each single-cell sample. **(D)** Bar graph illustrating the proportion of aneuploid and diploid cells in each single-cell sample. **(E)** Clustering and annotation of single-cell sequencing data on the basis of the results of TSNE downscaling. **(F)** Clustering and annotation of single-cell sequencing data on the basis of the results of UMAP downscaling. **(G)** Heatmap showing the expression of 9 tertiary lymphoid structure-related genes in different cell subpopulations.

To investigate the expression of 9 TLs-associated genes in the tumour microenvironment (TME) of HNSCC, we analysed the HNSCC single-cell dataset GSE103322. The dataset contains 7 major cell types, showing the distribution and quantity of different cell types (**Figures 2E, F**). CCR7 and CXCR4 were predominantly expressed in CD8+ T cells, with lower expression detected in other cell types. BCL6 presented relatively high expression levels across all the subpopulations. Interestingly, our findings revealed that all nine TLs-associated genes were associated with immune cell infiltration, underscoring the significant role of TLSs in the immune microenvironment of HNSCC (**Figure 2G**). The results from the copykat and cell cycle analyses were visualized via t-distributed stochastic neighbour embedding (t-SNE) plots (**Figures 2A, B**), which depicted the proportion of malignant and nonmalignant cells in each sample, as well as the distribution of cells across different cell cycle phases. These proportions are further illustrated via bar graphs (**Figures 2C, D**).

### 3.3 Identification of clusters of TLSs

**Figure 3:**
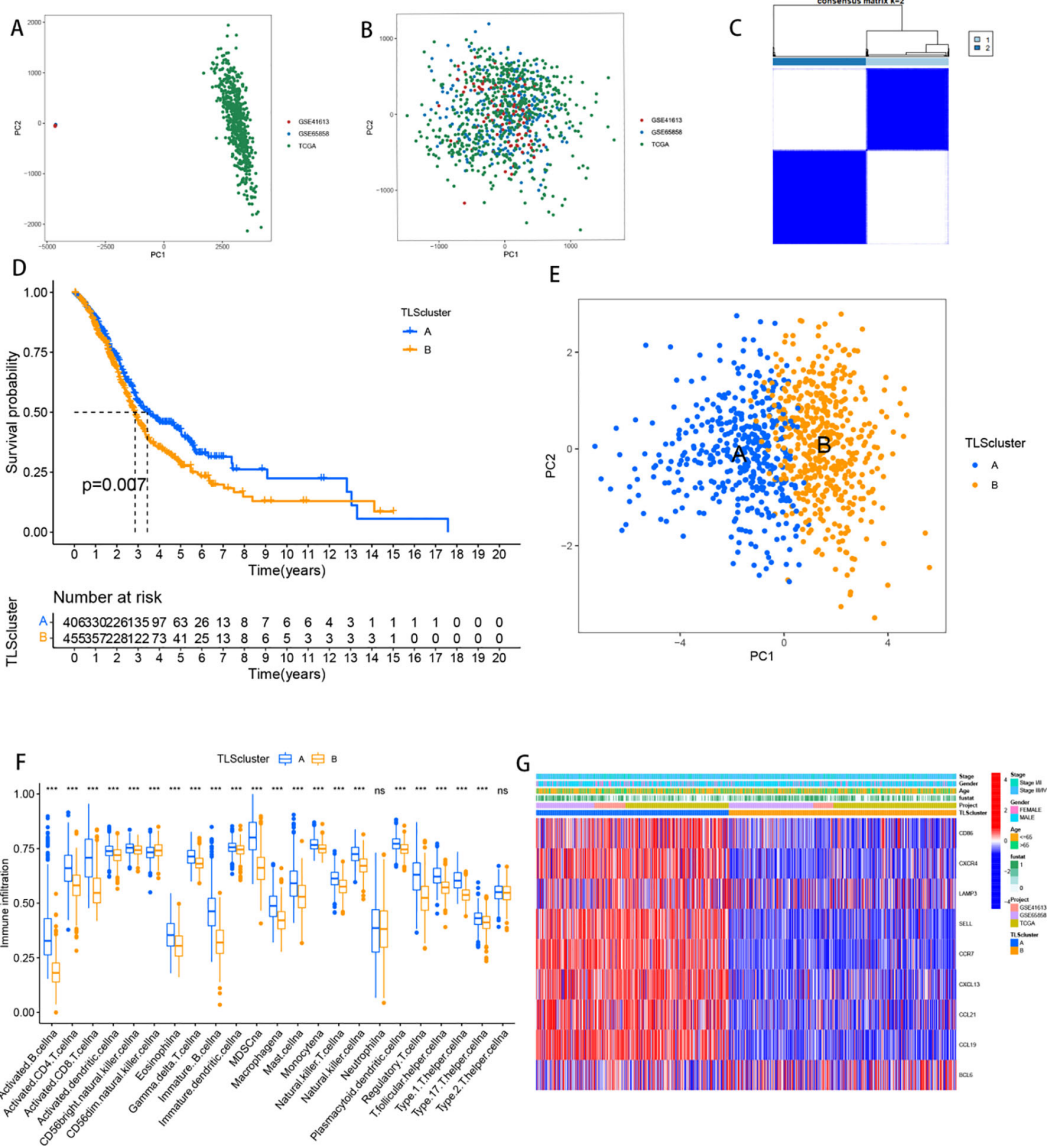
Identification of clusters of TLSs. **(A)** Dot plot of principal component analysis of TCGA and GEO transcriptome sequencing data prior to the removal of batch effects. **(B)** Dot plot of PCA results following the removal of batch effects, demonstrating improved sample clustering and reduced technical variability. **(C)** Heatmap displaying the consistency matrix for k = 2 clusters. Rows and columns represent samples, with color intensity (white to dark blue) indicating the likelihood of samples clustering together (0 = unlikely, 1 = always). **(D)** Kaplan-Meier curves comparing overall survival (OS) between the two patient subcategories. The horizontal axis represents survival time (OS), and the vertical axis represents survival probability. Descending curves indicate patient mortality, with the accompanying table showing the number of patients under follow-up at each time point. **(E)** Principal component analysis of two patient subclasses, with each point representing a sample. **(F)** Box plots of immune cell infiltration analysis for two patient subcategories; *** represents *p*<0.001, and ns represents *p*>0.05. **(G)** The expression levels of 9 TLS-associated genes in the two subgroups.

The combined TCGA-HNSCC, GSE41613 and GSE65858 cohorts were analysed via principal component analysis (PCA), which revealed a significant reduction in the corrected batch effect (**Figures 3A, B**). The results revealed that k=2 was the optimal number of clusters, with 869 HNSCC patients divided into 2 clusters (Cluster A: 410 samples; Cluster B: 459 samples) (**Figure 3C**). K−M survival analysis revealed that patients in Cluster B had a poorer prognosis than those in Cluster A (**Figure 3D**, p=0.007). Further PCA analysis revealed significant differences in the transcriptional profiles between the two clusters (**Figure 3E**). Exploration of the tumour microenvironment via the “ssGSEA” algorithm revealed differential enrichment of immune cells, with Cluster A showing a greater abundance of immune cells than Cluster B, except for natural killer cells, monocytes and CD4+ T cells (**Figure 3F**). A heatmap analysis revealed significant differences in TLS expression and clinicopathological features between the clusters, with significantly higher TLS expression in Cluster A (**Figure 3G**).

Subsequent differential expression analysis revealed 5501 DEGs between Clusters A and B. GO enrichment analysis revealed that these DEGs were involved in functions related to immune cell activation and substance transport pathways, such as the secretory granule membrane, leukocyte proliferation, regulatory effects on T cell activation, and positive regulation of cell−cell adhesion (**Supplementary Figure 2A**). Additionally, KEGG enrichment analysis revealed associations of these genes with the progression of certain viral bacterial infections and immune cell differentiation (**Supplementary Figure 2B**). **Supplementary Figure 2C** is an enrichment circle plot. From the outer to the inner circles: the first circle represents GO IDs, the length of the bars in the second circle corresponds to the number of background genes, with colour intensity indicating the significance level. The third circle corresponds to the number of target genes, and the fourth circle represents the enrichment factor.

### 3.4 Identification of TLS gene clusters

**Figure 4:**
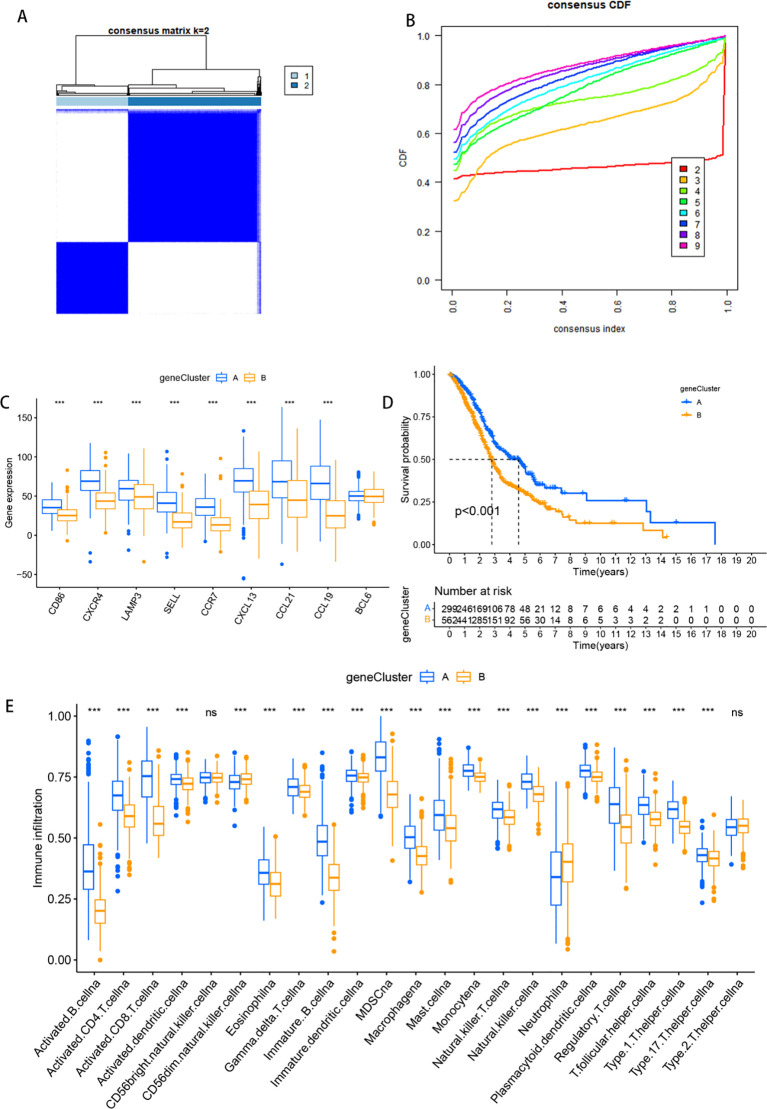
Identification of TLS gene clusters. **(A)** Matrix heatmap for k = 2: the rows and columns of the matrix represent the samples, the values of the consistency matrix are represented in white to dark blue on a scale from 0 (unlikely to cluster together) to 1 (always cluster together), and the long bar between the dendrogram and the heatmap is the classified category. **(B)** The CDF plot illustrates the cumulative distribution function for different values of k, reflecting the stability of clustering across varying numbers of clusters. **(C)** Box plots of the expression of nine tertiary lymphoid structure-related genes in different subclasses of patients. **(D)** Kaplan-Meier curves compare overall survival (OS) between the two patient subcategories. The horizontal axis represents survival time (OS), while the vertical axis represents survival probability. A descending curve indicates patient mortality, and the accompanying table provides the number of patients under follow-up at each time point. (OS) in the horizontal coordinate and survival rate in the vertical coordinate. The starting point is the time of the start of the value visit, a descending curve represents the death of the beneficiary, and the table indicates how many people were in follow-up at each time point. **(E)** Box plots of immune cell infiltration analysis for two patient subcategories; *** represents *p*<0.001, and ns represents *p*>0.05.

Among the 5501 DEGs, 1539 genes associated with the prognosis of HNSCC patients were identified through univariate Cox analysis. Clustering classification was subsequently conducted on these 1539 DEGs. When k=2, the PAC value reached its minimum, resulting in the division of 869 HNSCC patients into 2 gene clusters: gene Cluster A with 304 samples and gene Cluster B with 565 samples (**Figure 4A**). The heatmap in **Figure 4B** illustrates the expression of the 1539 DEGs in the two gene clusters, along with the clinicopathological characteristics of each sample. Notably, all nine TLs-associated genes were more highly expressed in gene Cluster A (**Figure 4C**). Furthermore, Kaplan−Meier survival analysis indicated a significantly better prognosis for patients in gene Cluster A than for those in gene Cluster B (**Figure 4D**, p < 0.001). To explore the tumour microenvironments of the two gene clusters, we used the “ssGSEA” algorithm. Consistent with the TLS clusters observed earlier, the differential enrichment of immune cells revealed a greater abundance of immune cells in Cluster A than in Cluster B, except for CD56bright natural killer cells and type 2 helper T cells (**Figure 4E**).

### 3.5 Development of a novel TLS scoring system

**Figure 5:**
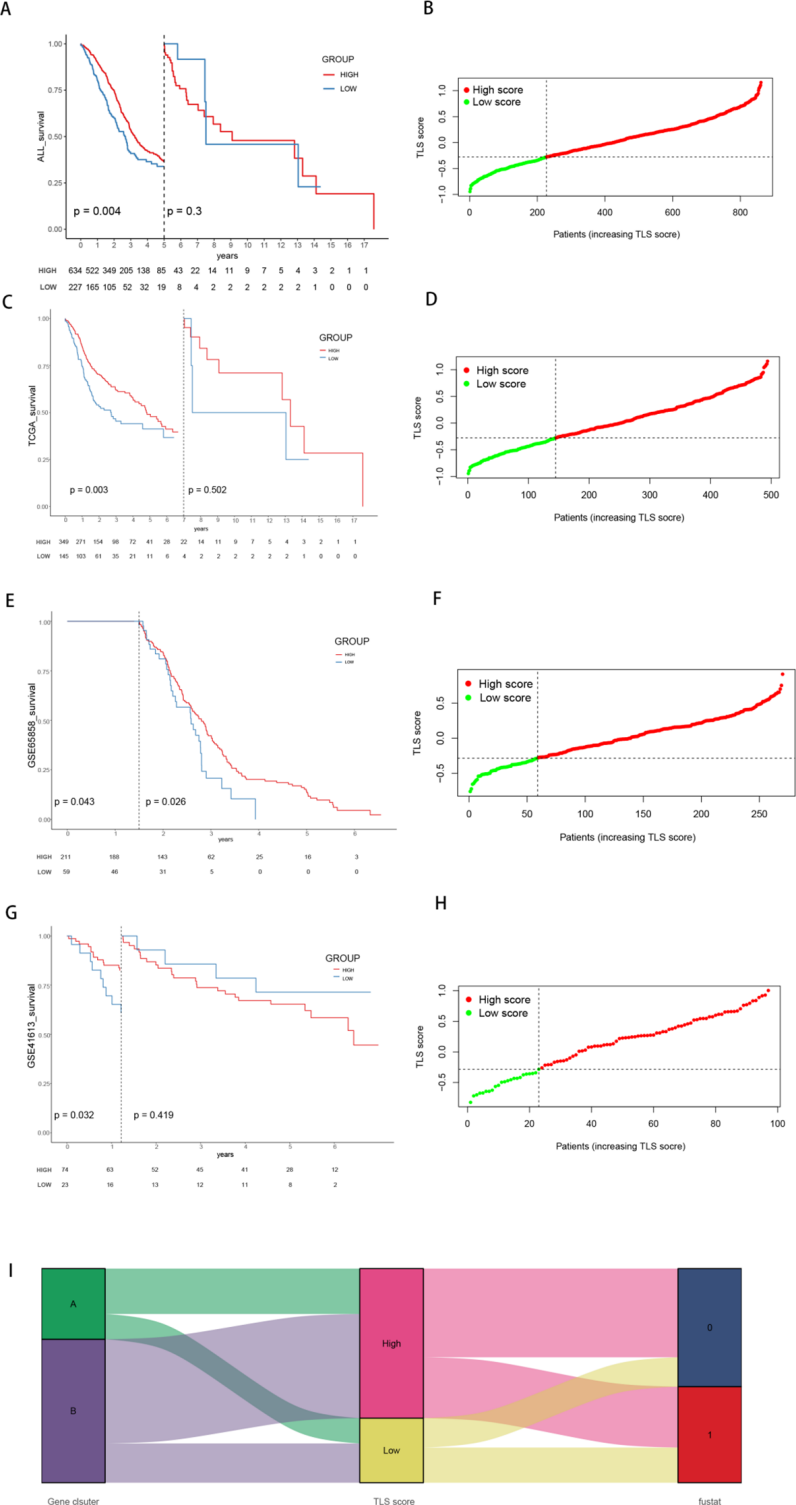
Survival curves and patient distributions of the high- and low-TLS score groups across the three cohorts. **(A, B)** Survival curves and the proportions of patients in the high- and low-score groups within the total cohort. **(C, D)** Survival curves and the proportions of patients in the high- and low-score groups within the TCGA cohort. **(E, F)** Survival curves and proportions of patients in the high- and low-score groups within the GSE65858 cohort. **(G, H)** Survival curves and proportions of patients in the high- and low-score groups within the GSE41613 cohort. **(I)** Sankey diagram illustrating the grouping of TLS scores by gene and survival status.

Among the 1539 DEGs, 980 genes positively associated with TLS gene cluster features were categorized as gene feature A, whereas 559 genes negatively associated with TLS gene cluster features were defined as gene feature B. The “Boruta” package was then used to run 500 iterations of the Boruta algorithm to further screen the candidate genes from the important genes. These genes were classified into three categories: confirmed, attempted and rejected. The TLSs of 869 HNSCC patients were subsequently estimated via the GSVA algorithm, and significant differences in OS were observed across the cohort and the three different cohorts (**Figures 5A, C, E, G**). Patients with higher scores had a potentially better prognosis. Score curves emphasized the strong discriminatory power of this TLS signature (**Figures 5B, D, F, H**). Furthermore, similar results were observed in the validation set GSE41116 (**Supplementary Figure 3B**). Moreover, Sankey plots revealed that a majority of the genes from TLS gene Cluster A with poorer prognoses belonged to the high TLS score subgroup, suggesting a better survival outcome (**Figure 5I**).

### 3.6 Relationships with the tumour microenvironment

**Figure 6:**
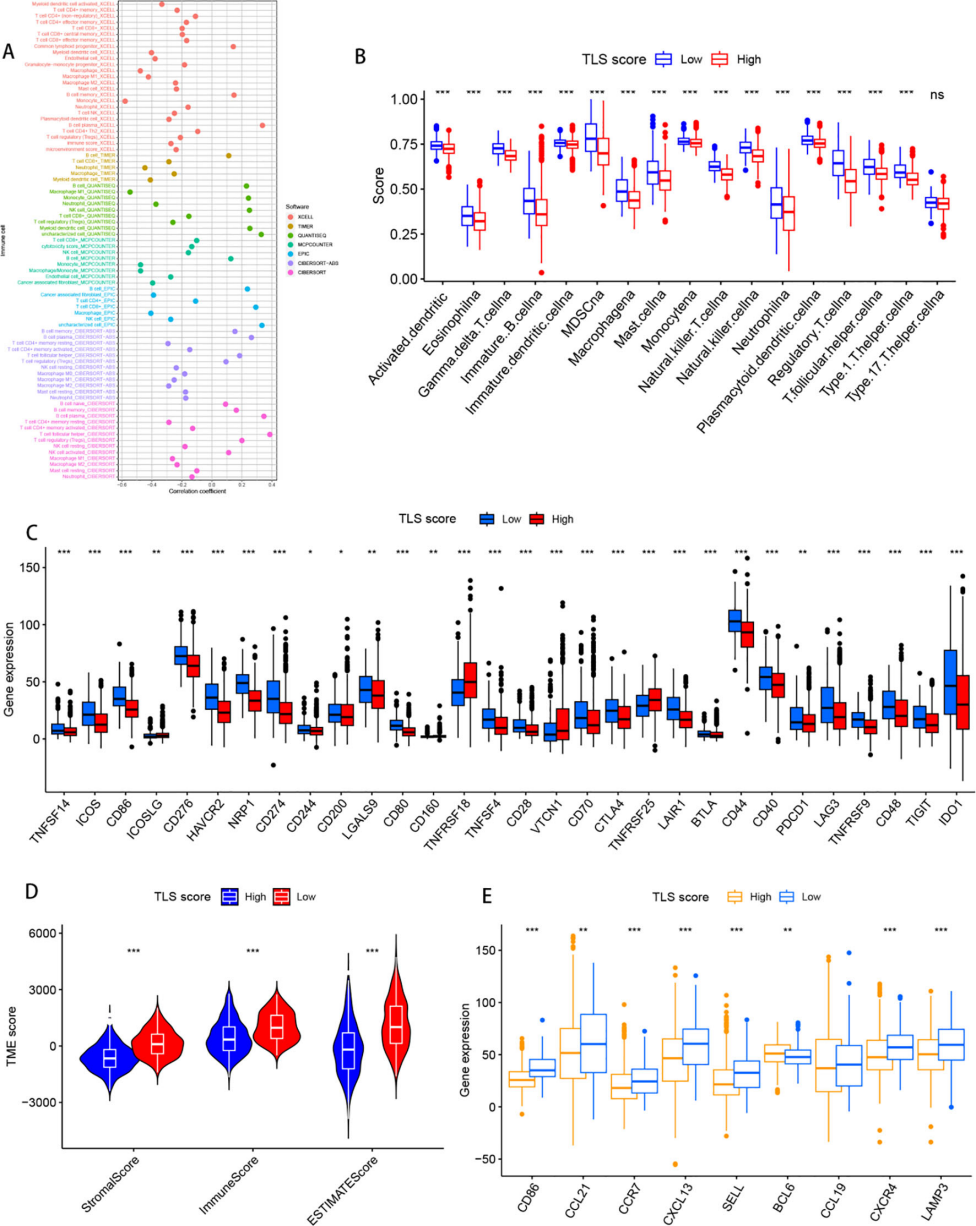
Relationships with the tumour microenvironment. **(A)** Bubble diagram of imputed immune cell infiltration in patients with tumours, with different colours representing different databases. **(B)** Degree of immune cell infiltration in both the high- and low-score clusters. p values are shown as follows: ns: not significant. **(C)** Degree of infiltration of tumour-associated immunity in both the high- and low-score clusters. **(D)** Relationships between the tumour microenvironment and immune risk characteristics. **(E)** Nine tertiary lymphoid structure-related genes expressed in patients with both high- and low-scoring clusters. *p< 0.05; **p< 0.01; ***p< 0.001, and ns represents *p*>0.05.

In the TCGA cohort, Spearman correlation was used to investigate the correlation between the TLS score and immune cell abundance in the HNSCC TME via various algorithms. The results revealed a negative correlation between the expression of most immune cells and the TLS score (**Figure 6A**). To further explore the correlation between TLS grouping and immune cells and functions, we quantified the ssGSEA enrichment scores of different immune cell subpopulations and related functions or pathways. The analysis revealed higher scores for eosinophils, immature B cells, immature dendritic cells, macrophages, mast cells, neutrophils, and regulatory T cells in the lower subgroups (**Figure 6B**). In addition, by comparing immune checkpoint activation between different risk groups, we found that almost all immune checkpoints, including HAVCR2, CD80, TNFSF4, and IDO1, were expressed at higher levels in the low-risk subgroup (**Figure 6C**). Similar findings were observed in the GSE41613 and GSE65858 cohorts. Additionally, the stromal score, immune score, and estimation score were greater in the low subgroup (**Figure 6D**). Additionally, the high-scoring group showed a higher tumour mutational burden (**Supplementary Figure 3A**) Notably, all nine TLs-associated genes were also more highly expressed in the low subgroup, suggesting that a higher level of immunity and immunogenicity in the TME is potentially influenced by tertiary lymphoid structures (**Figure 6E**).

### 3.7 Predicting and validating the efficacy of immunotherapies and predicting the effects of targeted chemotherapeutic agents

**Figure 7:**
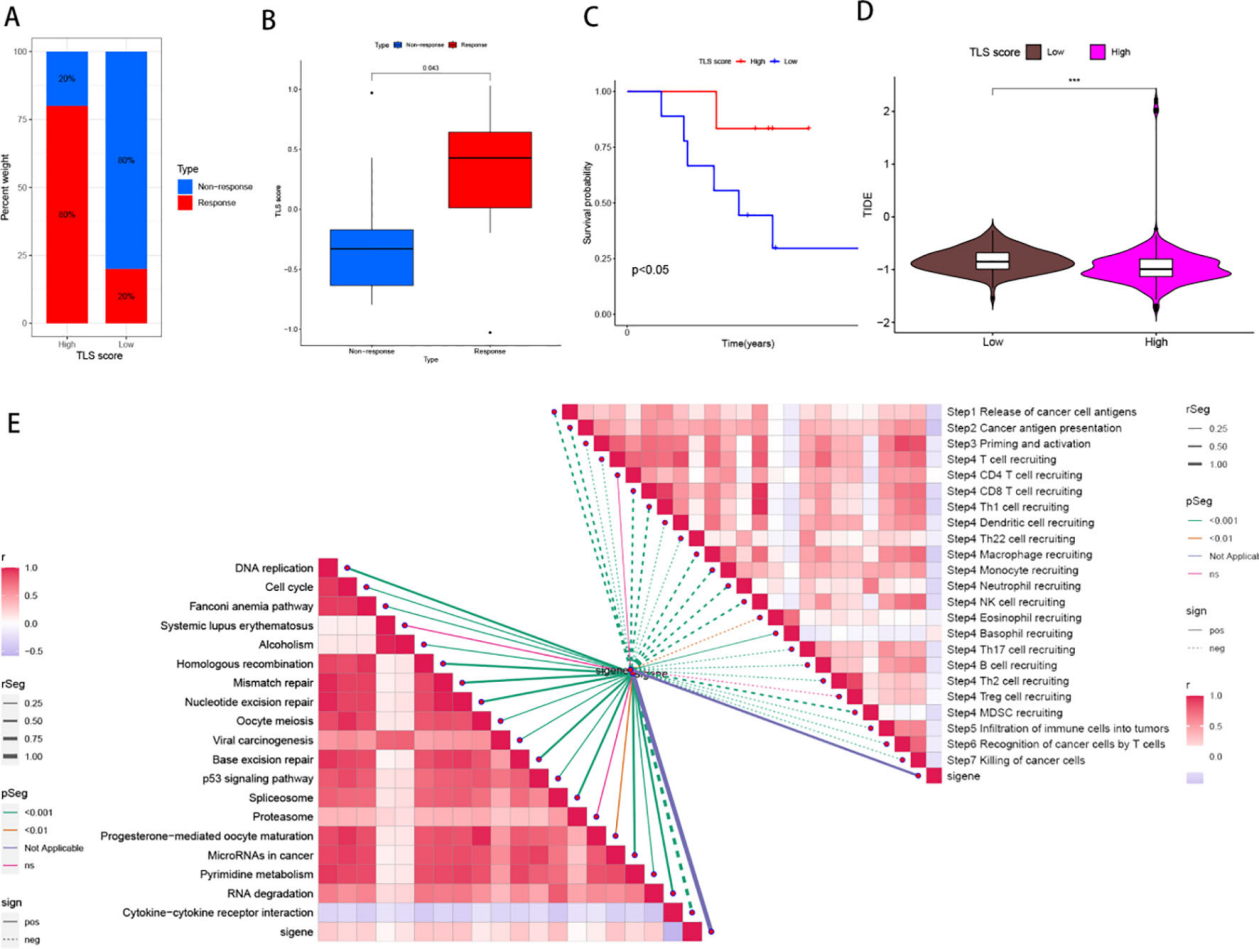
Predicting and validating the efficacy of immunotherapies and predicting the effects of targeted chemotherapeutic agents. **(A)** Bar graph of the proportion of patients in the high- and low-score clusters that responded to treatment in the sequencing data from the HNSCC immunotherapy cohort. **(B)** Box plots of model scores for patients who responded to treatment in sequencing data from the HNSCC immunotherapy cohort. **(C)** Survival analysis curves for patients with high and low scores for survival time (OS) in the horizontal coordinate and survival rate in the vertical coordinate. **(D)** Box plots of TIDE database scores for patients in the high and low subgroups of the HNSCC cohort in the sequencing data. **(E)** Correlations among TLS scores and immune checkpoint blockade therapy (ICB) response characteristics and tumour immune processes.

**Figure 8:**
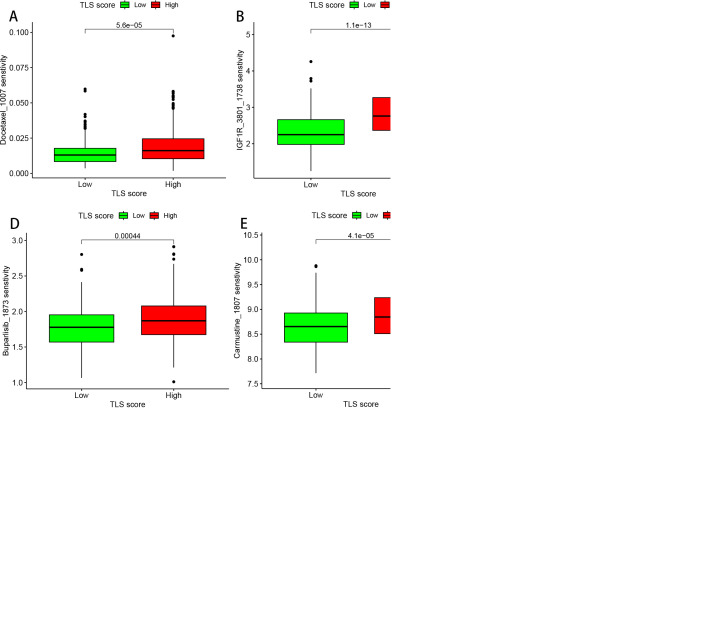
Drug sensitivity analysis of the TLS scoring system. **(A)** Box plot of the sensitivity of docetaxel_1007. **(B)** Box plot of the sensitivity of IGF1R_3801_1738. **(C)** Box plot of the sensitivity to docetaxel 1819. **(D)** Box plot of the sensitivity of buparlisib 1873. **(E)** Box plot of the sensitivity of carmine 1807. **(F)** Box plot of the sensitivity of cells to vincristine_1818. **(G)** Box plot of the sensitivity of teniposide 1809. **(H)** Box plot of the sensitivity of selumetinib 1736. **(I)** Box plot of the sensitivity of ULK1_4989_1733.

To test the predictive potential of our TLS profile in a real immunotherapy cohort, we selected a specific group of patients for analysis. The results revealed that patients who responded positively to immunotherapy had a greater effective response rate in the high-score group than in the low-score group (**Figures 7A, B**). Moreover, a large difference in OS was observed across the cohort (**Figure 7C**), suggesting that patients with higher scores may have a better prognosis. To rationalize which patients are more suitable for immunotherapy, we applied the TIDE score (TIDE score = T-cell dysfunction score - T-cell exclusion score) to assess immune function abnormalities in the tumour and regional lymph nodes. The results consistently indicated that patients in the higher subgroup had a greater probability of responding to immunotherapy (**Figure 7D**). The correlation between TLS scores and ICB-related positive signals was then further explored. The results revealed a positive correlation between the TLS score and various signals related to processes such as mismatch repair, the cell cycle, DNA replication, the depletion pathway, base excision repair, and microRNAs in cancer. The tumour immune cycle is a key indicator used to evaluate the biological function of the chemokine system and immunomodulators (24). Consequently, differences in the activity of tumour immune cycle steps between the high and low subgroups were shown, with most of the steps of the cycle showing upregulated activity, including the release of cancer cell antigens (step 1), presentation of cancer antigens (step 2), priming and activation (step 3), and entry of immune cells into the tumour (step 4). In addition, a more pronounced negative correlation was observed between each of these steps in the tumour immune cycle and the TLS score, as illustrated in **Figure 7E**. We subsequently analysed the impact of nine classical chemotherapeutic agents for head and neck cancer, including doxorubicin. Interestingly, all patients in the high-score subgroup had higher IC50 values than those in the low-score subgroup, suggesting that these high-score patients were more sensitive to these classical drugs (**Figures 8A–I**).

### 3.8 Validation of TLS signature expression at the cellular level

**Figure 9:**
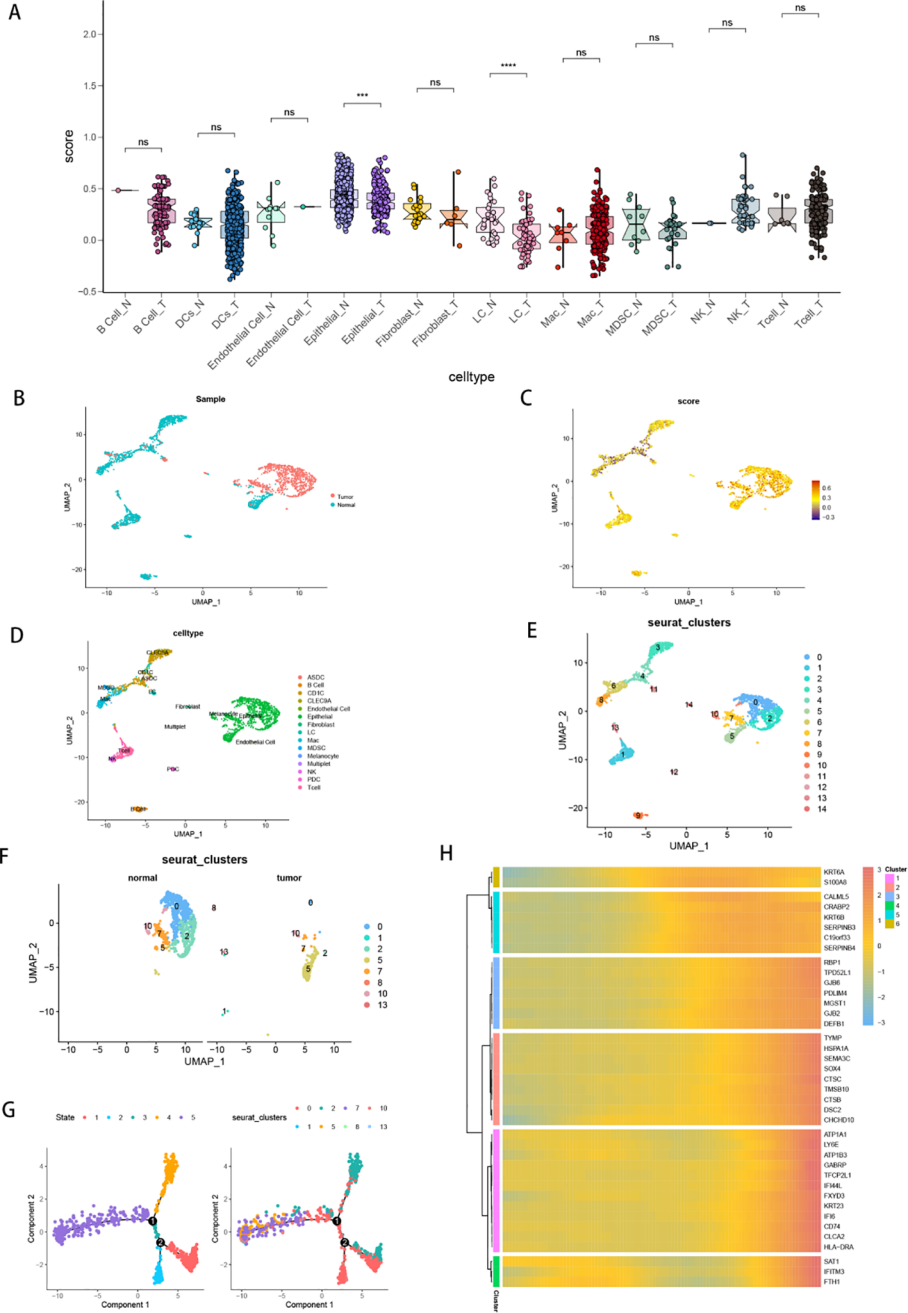
Cell TSL scoring and trajectory analysis. **(A)** Box plots comparing TSL scores between normal and tumour cell groups of different cell types; ***p<0.001, ****p<0.0001, and ns represents *p*>0.05. **(B)** UMAP plot of tumour cell groups versus normal cell groups. **(C)** UMAP plots of TSL scores. **(D)** UMAP plot with cell type annotations. **(E)** UMAP plot of cell clusters. **(F)** UMAP plot comparing epithelial cells in the tumour and normal groups. **(G)** Cell developmental trajectory plots of epithelial cells. **(H)** Pseudotemporal heatmap showing the specific expression of genes in the 5th cluster of epithelial cells.

A box plot comparison was conducted to illustrate the score disparities between the normal and tumour groups for different cell types, with the normal group exhibiting higher scores than the tumour group (**Figure 9A**). UMAP plots for the tumour and normal cell groups (**Figure 9B**), the TSL score UMAP plot (**Figure 9C**), the cell type annotation UMAP plot (**Figure 9D**), and the clustering UMAP plot (**Figure 9E**) collectively indicate higher scores in normal epithelial cells than in tumour cells. The UMAP plot results for tumour and normal epithelial cells revealed that Clusters 0 and 2 were primarily distributed in the normal group, whereas Cluster 5 was predominantly in the tumour group (**Figure 9F**). The cell development trajectory map (**Figure 9G**) showed that Clusters 0 and 2 were located mainly at the beginning of the cell trajectory, whereas Cluster 5 was situated primarily at the end of the cell trajectory. Additionally, the pseudotime heatmap of genes specifically expressed in Cluster 5 (**Figure 9H**) highlights the contribution of these genes to tumour cell development at different stages, further confirming that Cluster 5 predominantly consists of malignant tumour cells in the late stage of development.

### 3.9 Signal pathway enrichment of TLS epithelial cell subpopulations and distribution in idle data

**Figure 10:**
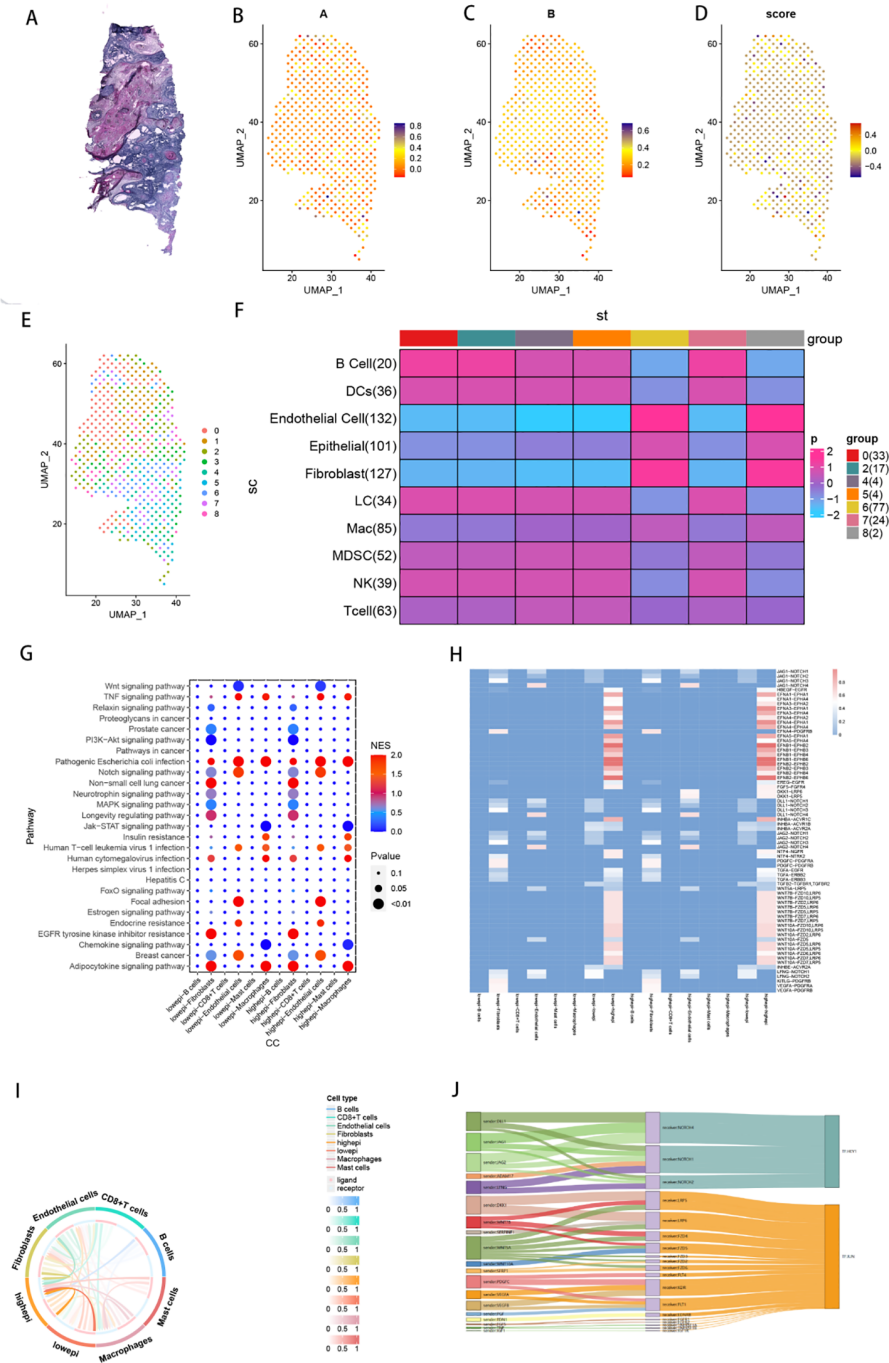
Cellular Communication Analysis. **(A)** Analysis of intercellular communication pathways between highepi and lowepi cells and other types of immune cells. **(B)** Heatmaps depicting the activity levels of intercellular communication between highepi and lowepi cells and other types of immune cells (standardized scores greater than 0.5). **(C)** Chord diagrams illustrating the intercellular communication between highepi and lowepi cells and other types of immune cells. **(D)** Sankey diagrams illustrating the intercellular communication between highepi and lowepi cells and other types of immune cells.

**Figure 10A** shows H&E-stained tissue sections from HNSCC patients. The enrichment scores of gene sets A and B, as well as the TLS signature, are displayed on the spatial transcriptomic data in **Figures 10B–D**. **Figure 10E** presents a UMAP plot illustrating the distribution of 9 subgroups in the spatial transcriptomic data. The results of multimodal intersection analysis (MIA) indicated that Groups 0, 2, 4, 5, and 7 presented the strongest correlation with epidermal cells and fibroblasts (**Figure 10F**).

On the basis of the median TLS score, epithelial cells in the tumour group were divided into high TLS score epithelial cells (high-TLS score epithelial cells) and low TLS score epithelial cells (low-TLS score epithelial cells). Intercellular pathway analysis between high-epithelial cells and low-epithelial cells and other immune cell types revealed that, compared with other immune cells, fibroblasts serve as the primary receivers of high-epithelial cells and low-epithelial cells (**Figure 10I**). Cell pathway activity analysis revealed that the Wnt signalling pathway (WNT2B-FZD1, LRP6), MAPK signalling pathway (EFNA4-PDGFRB, EGF7-FGFR1), EGFR tyrosine kinase inhibitor resistance, cellular senescence, and axon guidance (EFNB1-EPHB3) pathways were significantly enriched between epithelial cells and fibroblasts (**Figures 10G–H**). The transcription factors enriched primarily in cell pathway enrichment included CTNNB1, TCF7, TCF7L2, ATF2, and ABL1 (**Figure 10J**).

### 3.10 SLC5A8 knockdown promotes HNSC proliferation, migration and invasion

**Figure 11:**
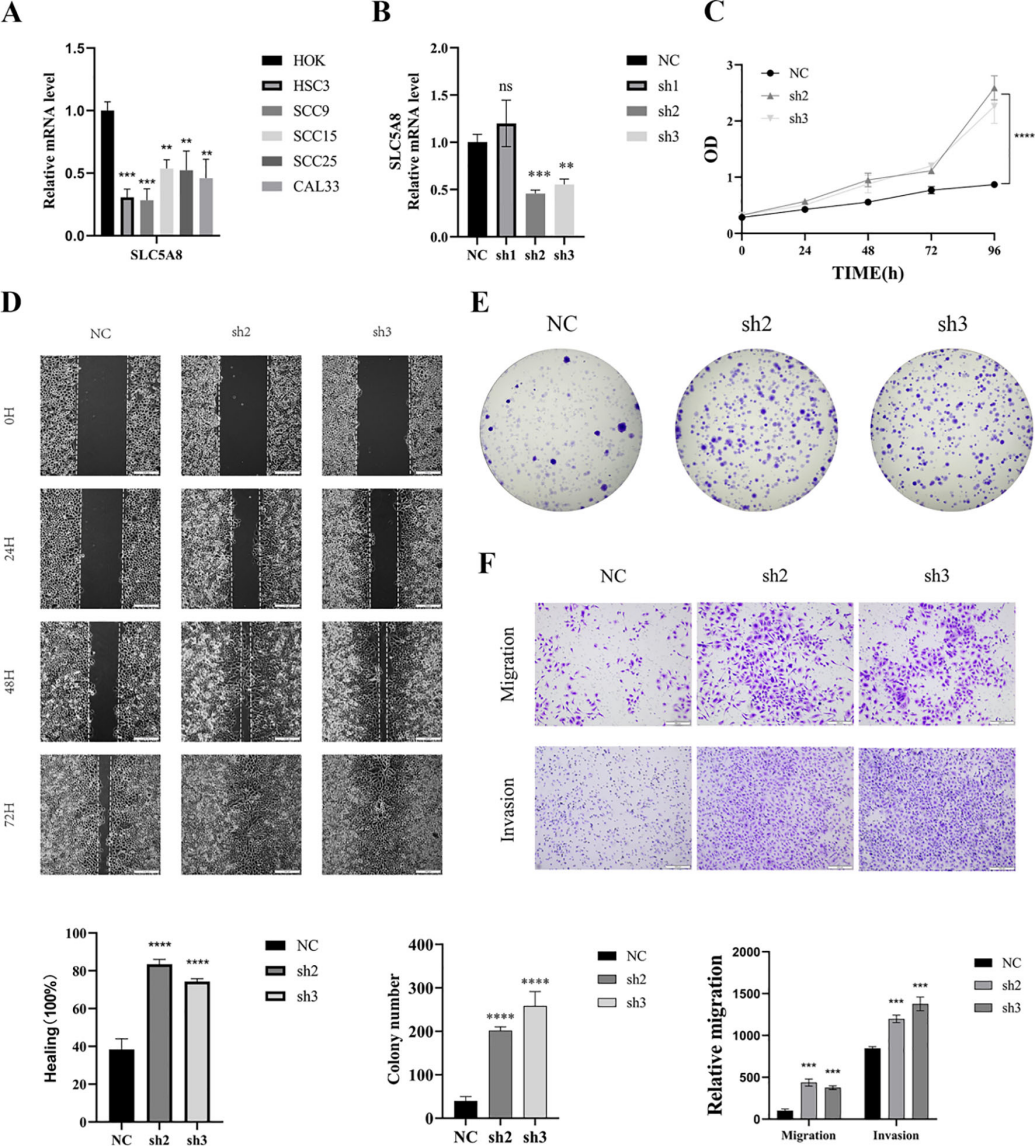
Knockdown of SLC5A8 promotes HNSC proliferation, migration, and invasion. **(A)** The mRNA expression levels of SLC5A8 in HOK and various HNSCC cells. **(B)** SLC5A8 knockdown effect was verified by qRT-PCR. **(C)** Knockdown of SLC5A8 promoted cell proliferation as shown by CCK-8 assay in SCC9 cells. **(D)** Knockdown of SLC5A8 promoted SCC9 cells migration capability as identified by wound healing assays. **(E)** The number of colonies increased dramatically after knockdown of SCL5A8 in SCC9 cells. **(F)** Transwell assays with SLC5A8 knockdown for SCC9 cells.

A comparative analysis of the impact of the expression levels of 12 genes within the signature on survival curves indicated that SLC5A8 expression had the most profound effect on patient survival (**Supplementary Figure 1A**). Moreover, in a combined analysis of SLC5A8 expression with tumour mutational burden (TMB), higher expression levels were associated with improved prognostic outcomes (**Supplementary Figures 1B-C**). To further investigate the function of SLC5A8 in HNSCC, we performed *in vitro* experiments to investigate its role in HNSCC cells. The qRT−PCR results revealed that SLC5A8 was expressed at significantly lower levels in HNSC (**Figure 11A**). The level of SLC5A8 expression was assessed 24 hours after transfection via qRT−PCR to evaluate the efficacy of siRNA-mediated SLC5A8 knockdown in SCC9 cells. The results showed that all three siRNA sequences successfully inhibited the expression of SLC5A8 mRNA, with siRNA-1 and siRNA-3 demonstrating stronger silencing effects; thus, these sequences were chosen for further experimentation (**Figure 11B**). Subsequent CCK8 assays revealed a significant increase in cell viability in SCC9 cells after SLC5A8 knockdown (P < 0.001) (**Figure 11C**). Similarly, scratch-wound healing assays demonstrated that wound healing was significantly accelerated after SCL5A8 was knocked down in cells (**Figure 11D**). Compared with those of the control cells, colony formation assays revealed a notable reduction in colony count in cells with reduced SLC5A8 expression, indicating a faster colony formation rate in SLC5A8-knockdown cells (**Figure 11E**). Additionally, transwell experiments revealed that SLC5A8 knockdown markedly increased the migration and invasion capabilities of SCC9 cells (**Figure 11F**).

## 4 Discussion

HNSCC consists of a group of malignant tumours of the upper aerodigestive tract (25), posing a significant global health challenge because of its high incidence and poor prognosis (26). Extensive research has led to the development of a multimodal treatment approach involving surgery, radiotherapy, chemotherapy, and targeted therapy, which has shown promising efficacy in early-stage HNSCC, with a 5-year survival rate of approximately 70%. However, the majority of patients are diagnosed at advanced stages because of the lack of obvious precancerous lesions (27). Despite established treatment strategies, challenges such as tumour drug resistance, local area infiltration, recurrence, and metastasis to neck lymph nodes continue to impact long-term survival, particularly in advanced cases (28). Therefore, there is an urgent need for the development of new therapeutic modalities to improve survival outcomes and quality of life for HNSCC patients. Recent studies have highlighted the importance of immune checkpoint blockade therapies in reactivating T cell responses to tumour cells, thus improving outcomes in cancer treatment (29–31). Specifically, in the case of HNSCC, pembrolizumab has shown superiority over standardized therapy or recurrent or metastatic cases (32), emphasizing the significant potential of immune checkpoint blockade therapy for the treatment of HNSCC. However, current TME staging systems for HNSCC are inadequate for assessing patient prognosis and the complex tumour microenvironment, highlighting the need for a new biomarker-based prediction system to guide clinicians in early intervention and personalized treatment strategies. The role of TLSs in oncology has gained significant interest, with evidence suggesting that TLSs contribute to generating and sustaining antitumour immune responses (33). TLS formation is closely related to the establishment of durable antitumour immunity (34), with studies in mouse models showing the role of IgG in initiating CD8+ T cell-mediated antitumour effects. In addition, B-cell-mediated antigen presentation in TLSs also induces the production of memory CD4 T cells (34). However, the specific mechanisms of action within TLSs and their effects on tumour development require further exploration through many basic experiments. TLSs have been proposed to stratify the prognosis of untreated cancer patients and serve as markers for evaluating the efficacy of immunotherapy (35). However, there is a lack of systematic studies in HNSCC. To address this gap, we integrated data from three HNSCC cohorts to construct a TLS-based scoring system for patient risk stratification. This system aims to assist physicians in devising effective immunotherapies and in better identifying individuals who may be more receptive to treatment.

In the present study, we collected data from three cohorts of HNSCC patients from various platforms, with 869 HNSCC patients being divided into two clusters. Given the important role of immune cell infiltration in the tumour microenvironment, we analysed the differences in the levels of various infiltrating immune cells between the two clusters. The results revealed that all immune cells, with the exception of neutrophils and type 2 helper T cells, infiltrated at a greater level in Cluster A than in Cluster B. Additionally, survival curves indicated that Cluster A, characterized by higher levels of immune infiltration, had a better prognosis. The subsequent investigations involved the extraction of DEGs from both clusters for subtyping. Once again, Cluster A patients had a better prognosis and a higher level of immune cell infiltration. More importantly, this study explored the impact of TLS-related features on the prognosis of HNSCC patients on the basis of the principle of the GSVA algorithm and used the Boruta algorithm to screen key genes and construct scores.

The tumour microenvironment is crucial in tumourigenesis, development, metastasis, and drug resistance. The immune microenvironment, an important component, has received significant attention. Studies have shown that HNSCC cells can directly promote monocyte CD16 expression *in vitro* (36), leading to monocyte differentiation into DC cells, which play pivotal roles in cancer immunotherapy (37). Additionally, macrophages have been shown to secrete VEGF, promoting neovascularization, tumour progression, and metastasis (38). The crosstalk between HNSCC cells and macrophages involves a paracrine angiogenic loop in which MCP-1 and TGF-β1 from HNSCC cells attract and activate macrophages to secrete TNF-α and IL-1, stimulating tumour cells to secrete more VEGF and IL-8 (39). High levels of macrophage infiltration in HNSCC patients correlate with increased extracapsular lymph node spread and lymph node metastasis (40). Therefore, future therapeutic strategies for HNSCC should focus on limiting the ability of tumour cells to recruit macrophages and reduce macrophage infiltration in the tumour microenvironment. In addition, TGF-β1 produced by tumour cells can also inhibit NK cell function by reducing the expression of NKG2D and CD16. In HNSCC, a cysteine-dependent apoptotic cascade in T lymphocytes can be triggered by tumour cells (41), leading to cell death. Regulatory T cells (Tregs) have been shown to induce Fas-mediated CD8+ T cell apoptosis, whereas CD4+ T cells are resistant to Treg-mediated Fas-mediated apoptosis. These studies exemplify the mechanisms of T cell apoptosis and depletion in HNSCC, although further mechanistic studies are still necessary for a more comprehensive understanding. In patients with HNSCC, tumours may achieve immune escape by reducing the number or altering the function of CD8+ T cells and CD4+ T cells. Furthermore, immune cell dysfunction in HNSCC patients is not limited to the tumour microenvironment, as functional defects and massive lymphocyte death have also been observed in the peripheral circulation of patients with advanced HNSCC (42).

Immunotherapy based on immune checkpoint inhibitors (ICIs) has become a crucial component of various cancer treatment strategies (43). Assessing immune checkpoint gene expression allows for the identification of HNSCC patients who may benefit more from immune checkpoint blockade therapy, enabling personalized precision therapy. In this study, we investigated the expression of immune checkpoints in two subgroups. The lower subgroup presented high expression levels of several immune checkpoints, including CTLA-4, CD44, CD276 and TNFRSF9. Among these, CTLA-4 plays a significant role in tumour cells through CTLA-4 to inhibit T cell activation and achieve immunomodulation (29). In HNSCC, Treg cells with high CTLA-4 expression actively proliferate and effectively suppress the immune response (44). Additionally, CTLA-4 competes with CD28 for binding to CD80/86, resulting in the direct inhibition of antigen presentation and T cell function (45). CD44-expressing HNSCC cells have a greater propensity for metastasis and inhibit the tumour killing effect of cytotoxic T lymphocytes (CTLs) by downregulating the Fas-FasL pathway (46, 47). CD276, which is highly expressed on the surface of HNSCC cells, helps tumour stem cells evade immune surveillance; blocking CD276 significantly inhibits lymph node metastasis and enhances antitumour immunity (43, 48). In combination with PD-L1, TNFRSF9 enhances tumour control by effectively activating and expanding the ability of specific cytotoxic T cells to target and eliminate tumour cells (49). Furthermore, CD200 can affect EMT gene expression and tumour regulation by binding to β-catenin and translocating it into the nucleus (50). IDO1 has been identified as a promising immune checkpoint target, as studies have shown that IFNγ suppresses the STAT1 signalling pathway and promotes the expression of IDO1, leading to the inhibition of cell death and the initiation of a dormant program (51). Additionally, LAG-3, a member of the immunoglobulin superfamily, is highly expressed on Tregs in HNSCC. Blocking LAG-3 has been shown to impede Treg activation and counteract Treg-mediated inhibition.

Reports suggest that CD80 competitively binds to PD-L1, inhibiting antigen presentation, and binds to CTLA-4, inhibiting T cell responses and collectively promoting immune escape (52, 53). We subsequently validated the accuracy and efficacy of the model in four patient cohorts receiving immunotherapy, which yielded highly satisfactory results. On the basis of the risk score, clinicians could assess the expression of immune checkpoints in patients, enabling the development of precise immunotherapy regimens and improving efficacy.

Among the signalling pathways regulated by low-epithelial and high-epithelial cells, ephrin-ephrin receptor signalling pathways, such as the EFNB2-EPHB2 and EFNA1-EPHA1 pathways, play a predominant role. The EphA1 receptor tyrosine kinase is implicated in cancer progression, tumour angiogenesis, and gynaecological diseases and is considered a driver gene in cancer genomics (54). Studies have indicated that the EFNA4-EPHA10 signalling cascade promotes cell migration and spheroid formation in oral squamous cell carcinoma cells, potentially impacting tumour growth and lymphatic metastasis (55). EphB4 knockdown has been shown to increase the expression of apoptotic proteins, including Bax and caspase-3, suggesting its potential role in promoting growth and invasion in head and neck squamous cell carcinoma. Patients who respond well to cetuximab, an EGFR-targeted therapy, tend to have low expression of EphB4 and high expression of ephrin-B2. Moreover, the overexpression of EphB4 and EFNB2 is associated with poor prognosis in head and neck squamous cell carcinoma patients and may serve as valuable prognostic markers (56).

SLC5A8, a conditional tumour suppressor, has been shown to have a protective effect against colitis and colon cancer in the context of a low-fibre diet. While this transporter is consistently expressed in normal colon tissues, it is silenced in colon cancer (57). The silencing mechanism is attributed to DNA methylation of the SLC5A8 promoter in both primary tumours and human colon cancer cell lines. Interestingly, inhibiting DNA methylation leads to an increase in the expression of SLC5A8 in colon cancer cells. Re-expression of SLC5A8 in colon cancer cells induces cell death, which is consistent with the characteristics of a tumour suppressor gene (58). Furthermore, SLC5A8 has been implicated in exerting tumour-suppressive effects in hepatocellular carcinoma by disrupting the Wnt/β-catenin signalling pathway.

## 5 Conclusion

In conclusion, we systematically studied TLSs in HNSCC and successfully developed a tertiary lymphoid structure-related signature that can accurately assess the prognosis and immune status of patients with HNSCC and help clinicians identify patients who may have a better response or resistance to immunotherapy and chemotherapy. Overall, our research has the potential to improve the development of patient-oriented treatment strategies for patients with HNSCC in the future.

## Data Availability

The original contributions presented in the study are included in the article/**Supplementary Material**. Further inquiries can be directed to the corresponding authors.
