# Supplementary material for: A novel tertiary lymphoid structure-associated signature accurately predicts patient prognosis and facilitates the selection of personalized treatment strategies for HNSCC
Source: Front Immunol. 2025 Mar 13;16:1551844. doi: 10.3389/fimmu.2025.1551844 (PMC11965918; doi:10.3389/fimmu.2025.1551844)
Supplement: Supplementary file 1 [file DataSheet1.docx]

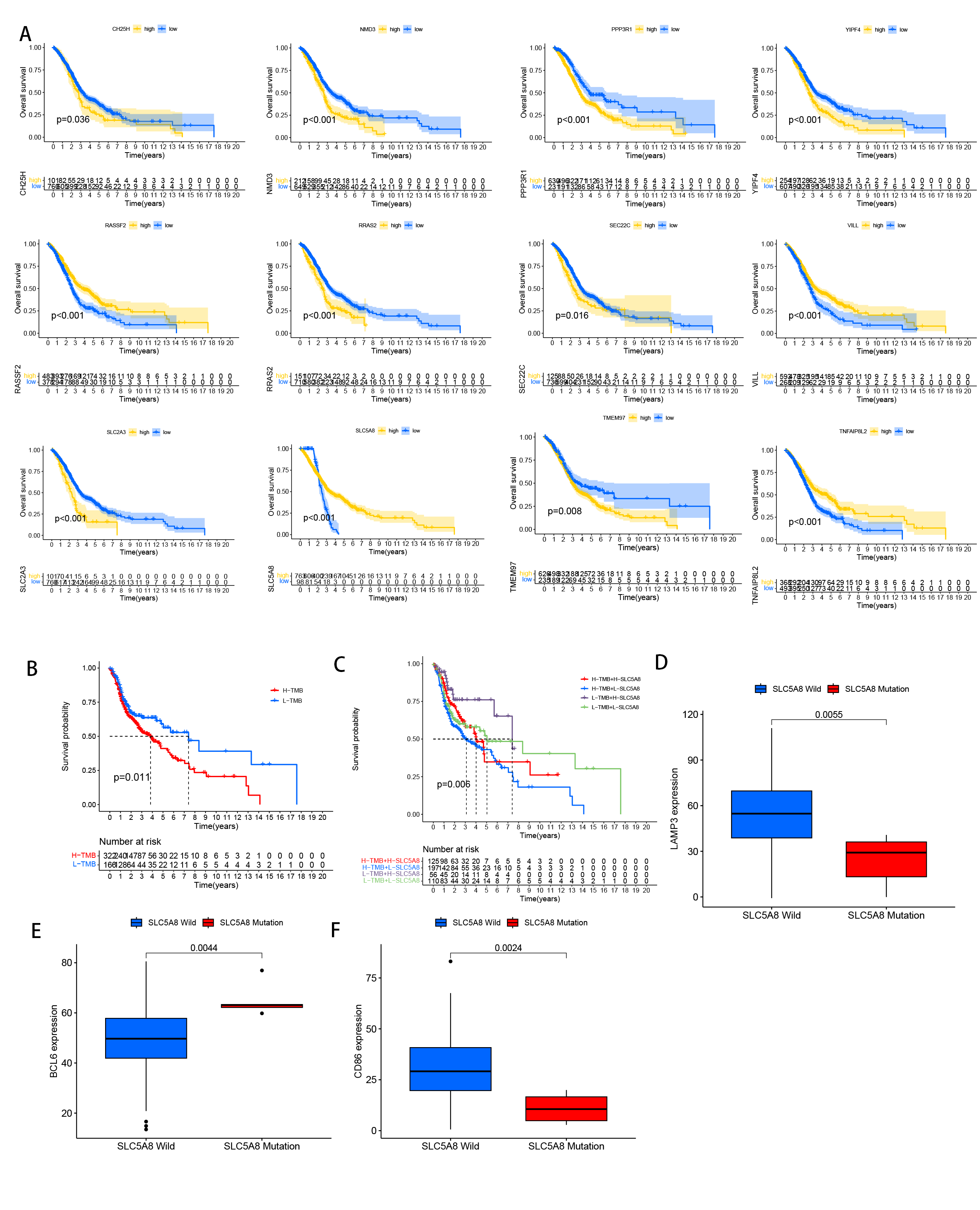


**Supplementary Figure 1**. Association of scoring signatures with prognosis and TMB. (A) Survival curves for 12 TLSs. (B) Survival curves for high and low TMB groups. (C) Survival curves for combined TMB and TLS grouping. (D) Expression levels of LAMP3 in SLC5A8 mutation and wild-type subgroups. (E) Expression levels of BCL6 in SLC5A8 mutation and wild-type subgroups. (F) Expression levels of CD86 in SLC5A8 mutation and wild-type subgroups.


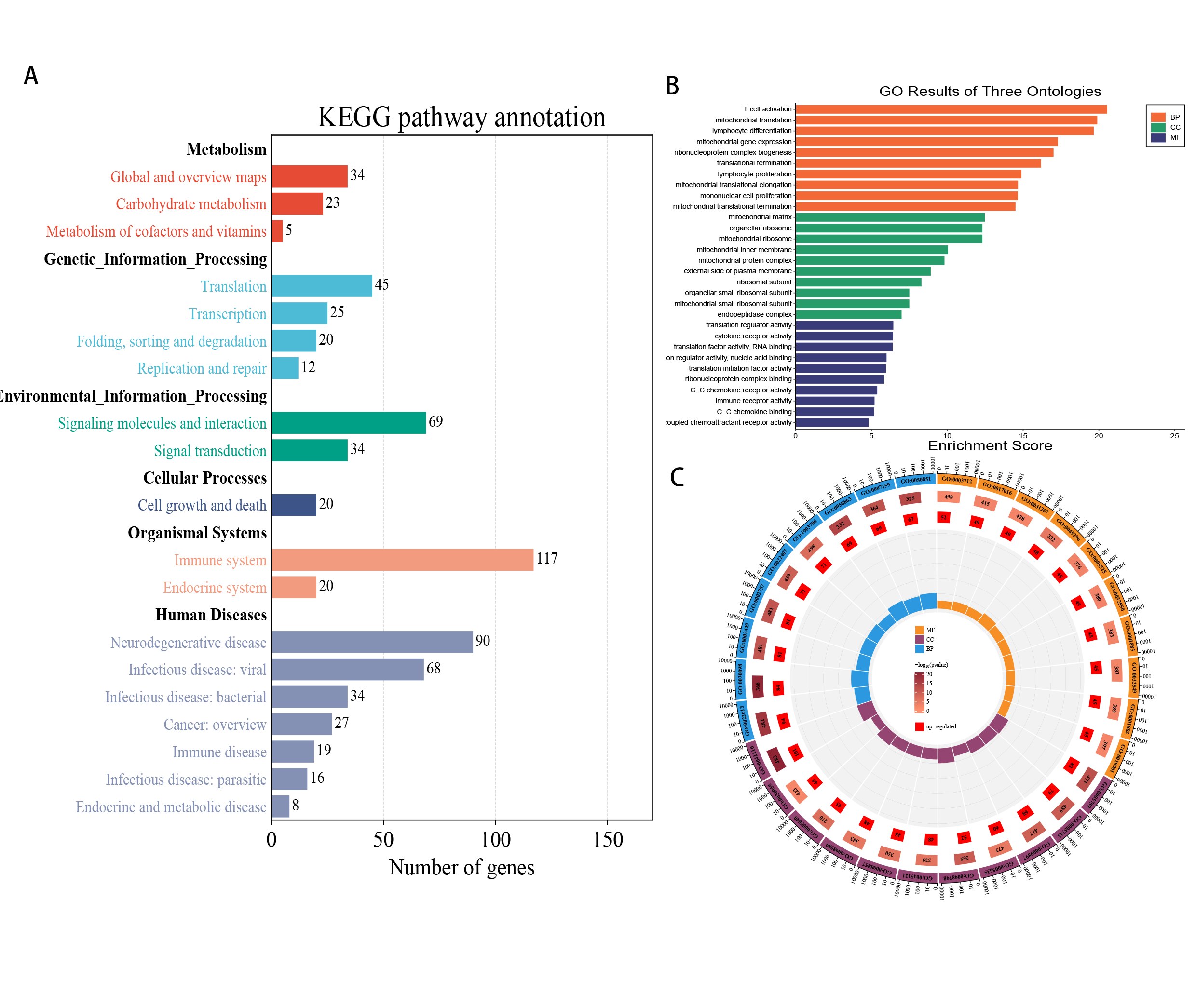


**Supplementary Figure 2.** Enrichment analysis of DEGs in clusters of TLSs. (A) Differential gene enrichment between the two patient subclasses out of the different KEGG subclass pathways, and the horizontal coordinate represents the number of genes that were enriched. Differential gene enrichment between two patient subclasses enriched with different GO subclasses, with the horizontal coordinate representing enrichment of the enrichment fraction (number of enriched genes/total number of pathway genes). (B) Bar chart. (C) Circos diagram.


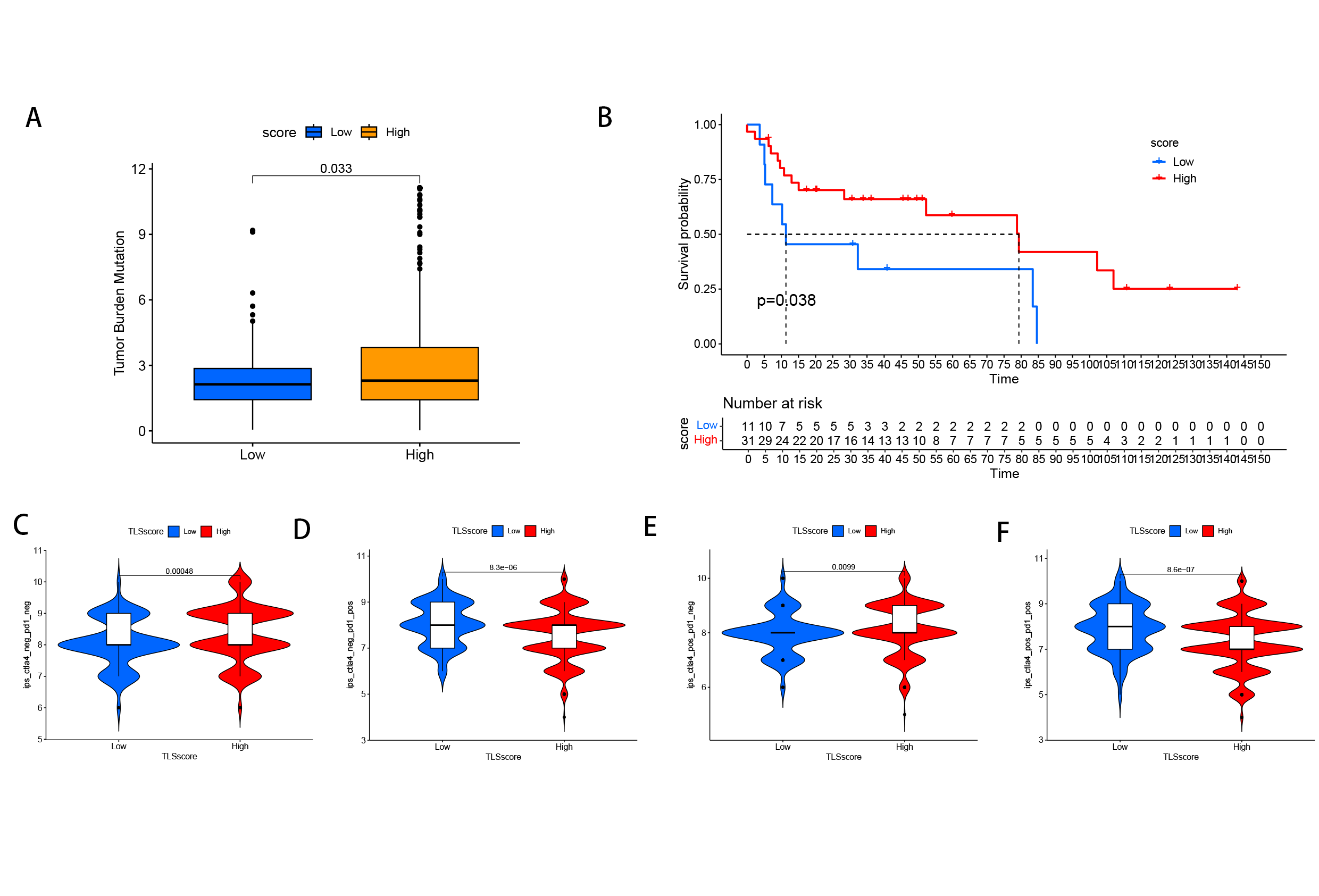


**Supplementary Figure 3.** (A) Differences in tumor mutational burden between high and low TLS score groups (B) Survival curve differences between high and low TLS score groups in the validation set GSE41116 (C) Differences between high and low TLS score groups in PD1-negative and CTLA4-negative responses (D) Differences between high and low TLS score groups in PD1-negative and CTLA4-positive responses (E) Differences between high and low TLS score groups in PD1-negative and CTLA4-positive responses (F) Differences between high and low TLS score groups in PD1-positive and CTLA4-positive responses
